# Expression of concern: Long non-coding RNA LINC00152 promotes gallbladder cancer metastasis and epithelial–mesenchymal transition by regulating HIF-1α via miR-138

**DOI:** 10.1098/rsob.230009

**Published:** 2023-01-18

**Authors:** Qiang Cai, Zhenqiang Wang, Shouhua Wang, Mingzhe Weng, Di Zhou, Chen Li, Jiandong Wang, Erzhen Chen, Zhiwei Quan

*Open Biol.* **10**, 20170247. (Published online 1 January 2017) (doi:10.1098/rsob.160247)

Subsequent to the publication of ‘Long non-coding RNA LINC00152 promotes gallbladder cancer metastasis and epithelial–mesenchymal transition by regulating HIF-1α via miR-138’ *Open Biol.* **10**, 20170247. (Published online 1 January 2017) (doi:10.1098/rsob.160247) it has been brought to the attention of the Editorial team that there are concerns regarding the validity of the data used in the study.

We are therefore issuing an expression of concern and will notify readers as to the results of our investigation as soon as possible.

Prof. Jon Pines FRS

Editor-in-Chief, Open Biology

